# Bilateral Virchow nodes: an unusual finding of pulmonary small-cell neuroendocrine carcinoma metastasis

**DOI:** 10.4322/acr.2023.455

**Published:** 2023-11-13

**Authors:** Matthew J. Zdilla, Alexander R. Gross, Tara Hajarat, Jeffrey A. Vos

**Affiliations:** 1 West Virginia University School of Medicine, Department of Pathology, Anatomy, and Laboratory Medicine, Morgantown, West Virginia, USA

**Keywords:** anatomy, carcinoma, lymph nodes, neoplasm metastasis, neoplasms

## Abstract

An enlarged left-sided supraclavicular node is a signal node for cancer metastasis. In such a case, the enlarged lymph node is often referred to as a Virchow node. The left-sided nature of the node is due to the drainage of the thoracic duct. So, the enlargement of a Virchow node is typically associated with malignancies, including gastrointestinal, pulmonary, and genitourinary carcinomas, in addition to lymphomas. This report documents a particularly unusual finding: bilateral Virchow nodes, representing metastasis of small-cell neuroendocrine carcinoma.

## INTRODUCTION

*Virchow node*, *Troisier node*, and *Virchow-Troisier* *node* are synonymous eponyms referring to a left-sided supraclavicular lymph node that is enlarged and palpable due to the metastatic spread of cancer. The visible appreciation of such a node (referred to hereafter as a Virchow node— the most common appellation), is known as a *Troisier sign.* The aforementioned eponyms are attributable to Rudolf Ludwig Karl Virchow and Charles-Emile Troisier, who identified the enlarged left supraclavicular lymph node as a sign of metastasis from abdominal cancers in the mid-to-late 19^th^ century. ^1,2,3^

From an anatomical perspective, the Virchow node is unique due to the asymmetrical territories drained between the right lymphatic and thoracic ducts. The latter, located on the left, drains the majority of thoraco-abdomino-pelvic organs via the left supraclavicular nodes. Thus, the Virchow node typically signals metastasis from the gut, ^1,2^ esophagus,^4^ liver,^5^ biliary tree,^6^ pancreas,^7^ urogenital organs,^3,8^ and lungs.^9^ The Virchow node has also signaled lymphoma,^10^ squamous cell carcinoma,^8^ and active tuberculosis.^11^

The Virchow node may also cause mass effects such as Horner syndrome.^12^ Due to its anatomical relationships with nearby anatomical structures, it has been posited that the Virchow node may be implicated in left-sided brachial plexopathy and decreased blood flow into the left upper extremity and, therefore, neurogenic and vascular thoracic outlet syndromes.^9^ Similarly, the Virchow node has also been suggested to be a possible cause of unilateral phrenic neuropathy.^9^

The Virchow node is a particularly important signal node of clinical significance for various of medical specialties. Further, the anatomical relationship between the Virchow node and local structures is clinically important, particularly with regard to potential mass effects. Thus, bizarre manifestations of the Virchow node warrant particular attention. This report documents a never-before-described case of bilateral Virchow nodes resulting from small-cell neuroendocrine carcinoma metastasis.

## CASE REPORT

Bilateral Virchow nodes were observed during the neck dissection of a 68-year-old white female donor body, whose cause of death was recorded as respiratory arrest secondary to lung cancer. The body donor willingly donated their body to advance of science through the West Virginia University Human Gift Registry. A formal autopsy was not performed on the donor body, and dissection was performed post-embalming. The donor body was located at the gross anatomical laboratory at West Liberty University. The West Virginia Anatomical Board approved the research.

## AUTOPSY PRESENTATION

**Figure 1:**
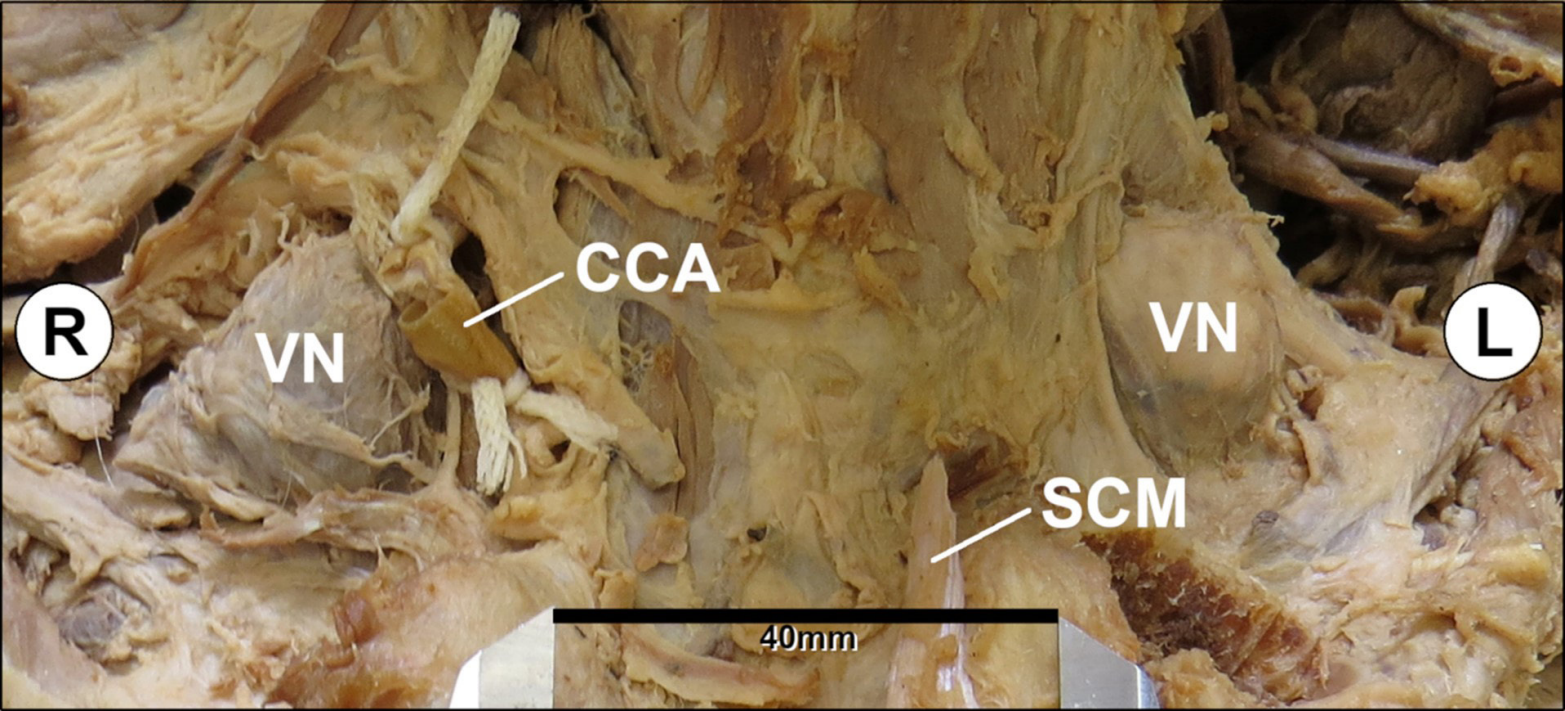
Anterior view of bilateral supraclavicular regions with sternocleidomastoid muscles reflected and bilateral Virchow nodes exposed. (**CCA:** right common carotid artery that has been cut and tied for the purpose of embalming; **SCM:** remnant of sternal head of left sternocleidomastoid; **VN:** Virchow node).

Dissection revealed bilateral level IV supraclavicular lymph nodes at the superior jugulosubclavian venous junction in the lesser supraclavicular fossa deep to the clavicular heads of the sternocleidomastoid muscles (Figure 1).

Each node was measured via digital calipers. The left Virchow node was 3.5 × 2.4 × 1.9 cm, and the right Virchow node was 3.7 × 3.6 × 1.8 cm.

**Figure 2:**
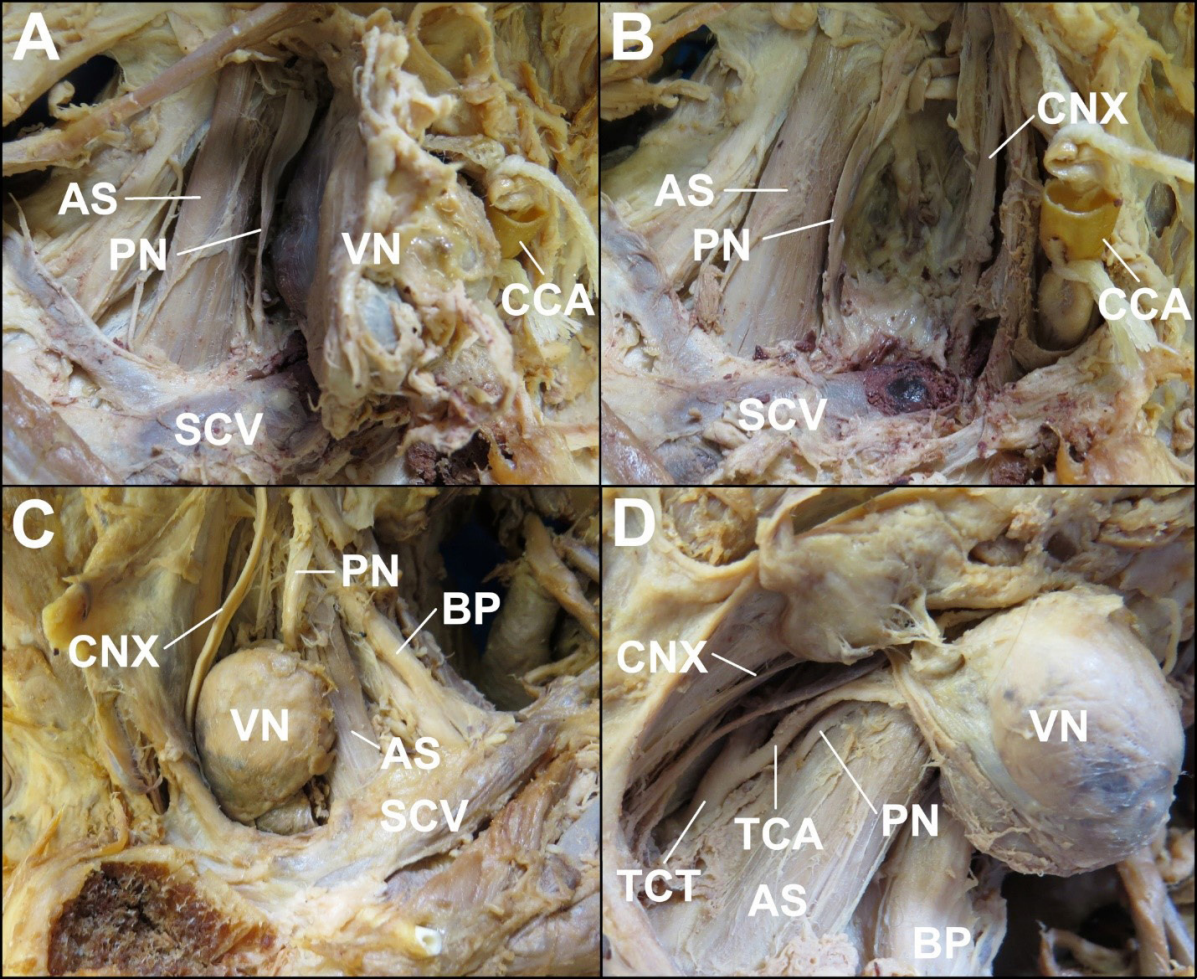
Anterolateral views of right- and left-sided supraclavicular regions (A&B and C&D, respectively) regions with sternocleidomastoid muscles reflected and bilateral Virchow nodes exposed or resected to reveal regional anatomical structures. **A –** Right Virchow node reflected anteriorly. The right phrenic nerve lies intermediate to the anterior scalene muscle and the Virchow node and the subclavian vein is located posterior to the inferior aspect of the Virchow node. **B –** With the right Virchow node resected, the vagus nerve is identified where the anteromedial aspect of the Virchow node would have otherwise been located. **C –** The left Virchow node is seen intermediate to the vagus nerve and phrenic nerve. The anterior scalene separates the Virchow node from the brachial plexus. **D –** With the left Virchow node reflected laterally the vagus and phrenic nerves are identified along the anterior and posterior boundaries of the fossa in which the Virchow node resided. Also seen are vascular structures including the thyrocervical trunk, located medial relative to the *in situ* position Virchow node and the transverse cervical artery, located posterior to the Virchow node and partly contained within the connective tissue upon the surface of the node (**AS:** anterior scalene muscle; **BP:** brachial plexus; **CCA:** right common carotid artery that has been cut and tied for the purpose of embalming; **CNX:** vagus nerve; **PN:** phrenic nerve; **SCV:** subclavian vein; **TCA:** transverse cervical artery; **TCT:** thyrocervical trunk; **VN:** Virchow node).

From a non-metric perspective, regional anatomical relationships were similar side-to-side. Each Virchow node was located anterior to the phrenic nerve, transverse cervical artery (which was involved within the connective tissues surrounding the right Virchow node), and anterior scalene muscle and posterolateral to the vagus nerve (Figure 2). The thyrocervical trunk and common carotid arteries were medial to the Virchow node (Figure 2).

**Figure 3:**
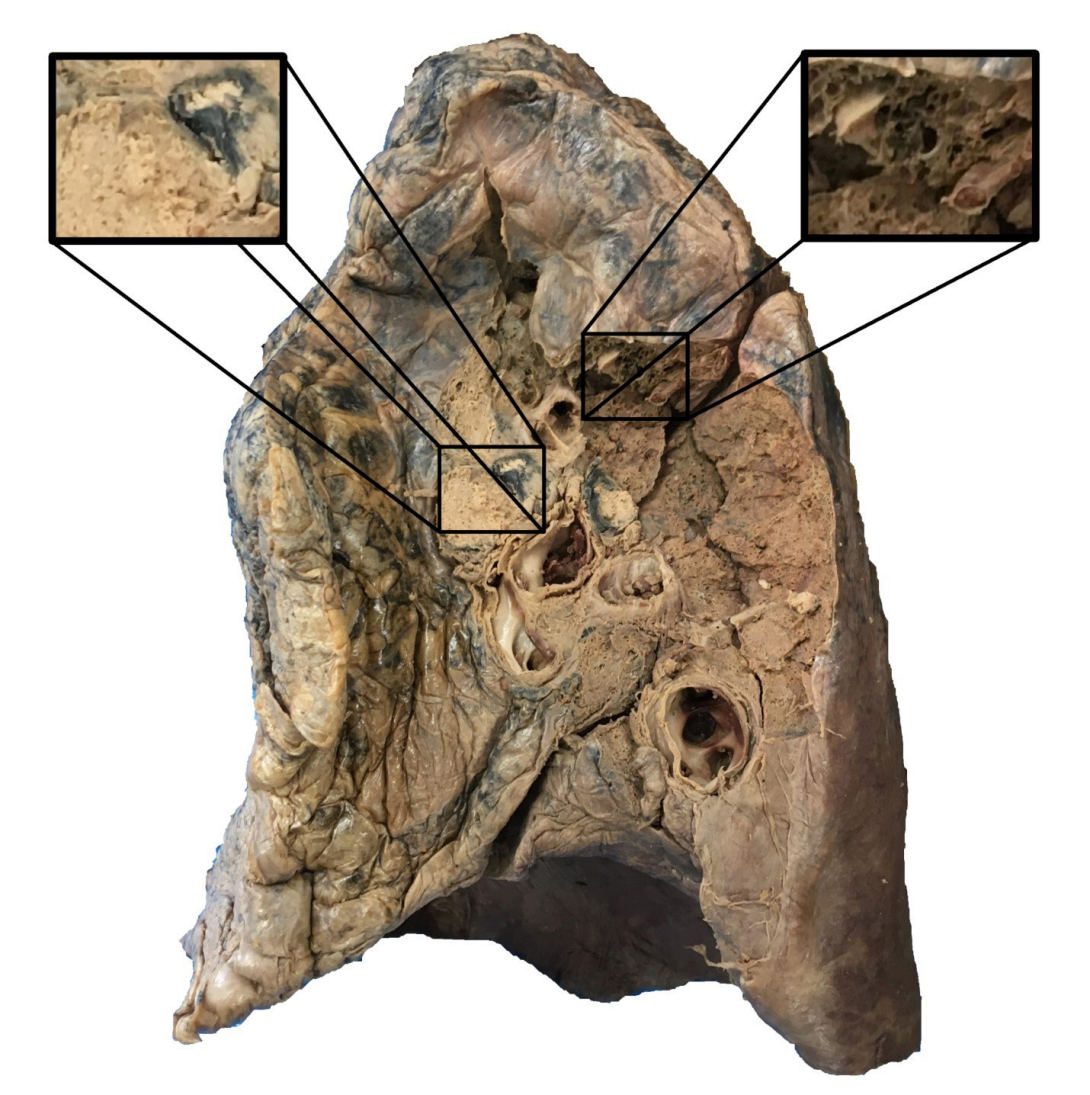
Hilar tumor involving the right lung. Also, anthracosis and emphysema are evident.

Beyond the local, regional anatomy, the hilum of the right lung was involved extensively by a tumor. The lung tissue was also to have emphysema (Figure 3).

**Figure 4:**
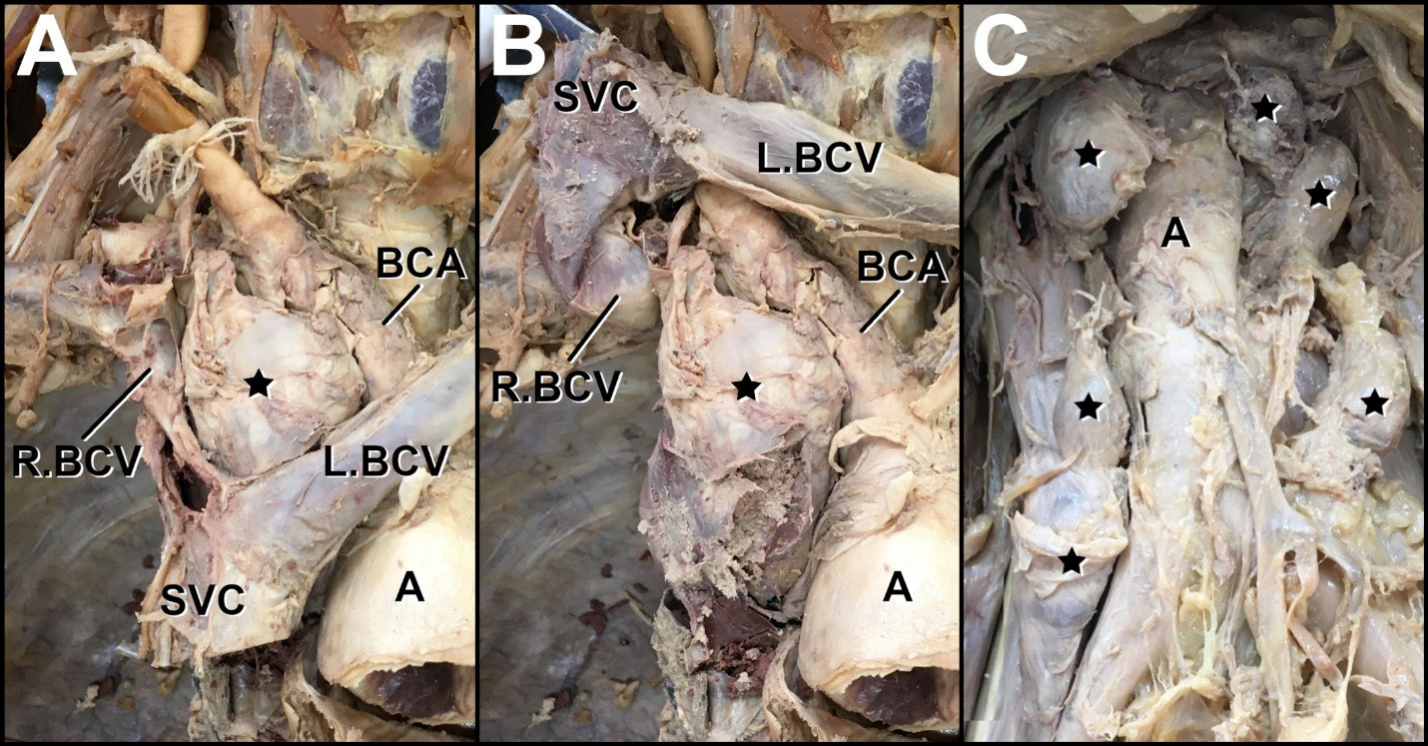
Non-Virchow nodal involvement. **A –** Pathologically enlarged lymph node located at the junction of the brachiocephalic veins, posterior to the superior vena cava, anterolateral to the brachiocephalic artery. The node is also positioned in the superior mediastinum medial to the lung and inferior to the right-sided Virchow node. **B –** Reflection of the superior vena cava and brachiocephalic veins reveals a node of particularly large size. **C –** Para-aortic “midline” adenopathy, unusual for carcinoma metastasis, rather typical of lymphoma. (**A:** aorta; **BCA:** brachiocephalic artery; **L.BCV:** left brachiocephalic vein; **R.BCV:** right brachiocephalic vein; **SVC:** superior vena cava; **STAR:** enlarged lymph node).

Hilar and mediastinal lymph nodes were enlarged and encroached upon local venous structures, including the superior vena cava and the brachiocephalic veins (Figure 4). Within the abdominal cavity, para-aortic “midline” adenopathy was also evident (Figure 4). All enlarged lymph nodes were similarly firm and rubber-like upon palpation; however, palpation was performed post-embalming and after hard fixation. Other gross findings included evidence of a total hysterectomy with bilateral salpingo-oophorectomy.

Histological examination of both Virchow nodes revealed interconnecting tumor nests in a predominantly trabecular pattern. Tumor cells showed hyperchromatic nuclei displaying powdery chromatin with a “salt and pepper” appearance and abundant mitoses.

**Figure 5:**
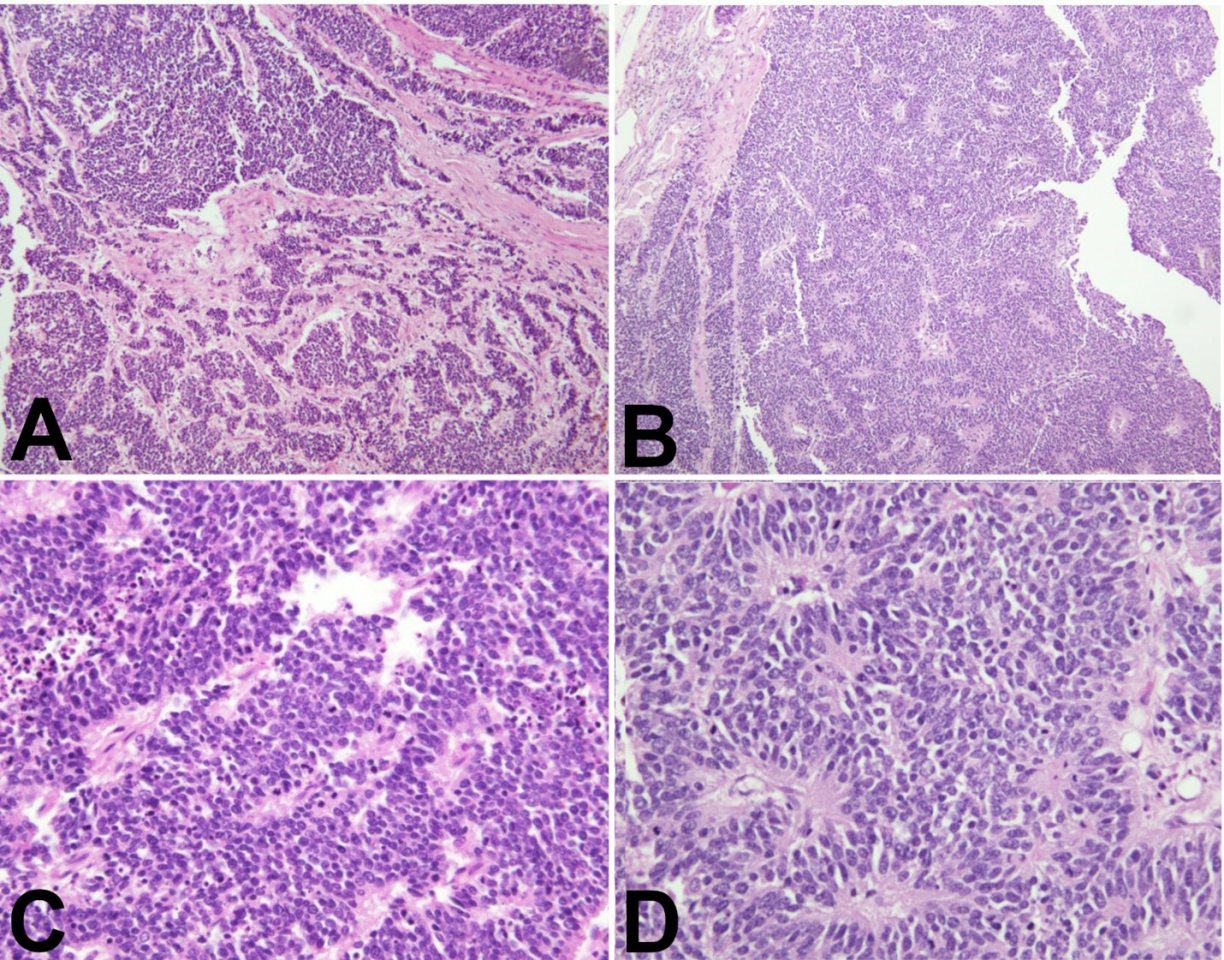
Photomicrograph compilation of H&E-stained Virchow node sections at 100× (**A&B**), and 400× (**C&D**). **A –** Interconnecting nests of hyperchromatic cells in a trabecular pattern. **B –** Hyperchromatic cells displaying multiple characteristic rosettes, suggestive of small cell neuroendocrine carcinoma. **C –**Sheet of cells with hyperchromatic nuclei with powdery chromatin with admixed necrosis and numerous mitotic figures, suggestive of small cell neuroendocrine carcinoma. **D –** Hyperchromatic tumor cells arranged in spoke-and-wheel configuration around central cores comprised of cytoplasmic processes. The rosettes are a feature of small cell neuroendocrine carcinoma.

Multiple characteristic rosettes were identified - cells in a spoke-and-wheel configuration around central cores comprised of cytoplasmic processes (Figure 5). The findings were consistent with Virchow nodal involvement by small-cell neuroendocrine carcinoma metastasis.

A systematic review of the literature was conducted to assess the prevalence of similar case presentations. The MEDLINE database was searched via the PubMed search engine. The following search terms were utilized: “bilateral” in combination with “supraclavicular lymph” and “Virchow” while excluding “Virchow Robin.” These terms were searched in both the title and abstract of MEDLINE records. Accordingly, the script utilized in the search read as follows:((((bilateral[Title/Abstract]) AND (supraclavicular lymph[Title/Abstract])) OR (bilateral[Title/Abstract])) AND (Virchow*[Title/Abstract])) NOT (Virchow robin). The search was performed for all articles *ab initio* until November 4, 2022.

A total of 21 search results were then screened for relevance. None documented enlarged bilateral supraclavicular lymph nodes and nore provided any gross anatomical details; however, one report by Fernández Aceñero et al.^13^ reported bilateral supraclavicular metastatic involvement by fine-needle aspiration cytology in three of 95 supraclavicular fine-needle aspiration cytology cases (57 of the 95 cases involved metastasis) assessed from seven years. The aforementioned cases included two cases of adenocarcinoma spread from the lungs and one from the large intestine.^13^

## DISCUSSION

The *Virchow node* is classically defined as an enlarged left-sided supraclavicular lymph node; however, occasionally, an enlarged right-sided lymph node has been referred to as a *Virchow node*. There have not been reports of concurrent bilateral “Virchow nodes”; thus, this report describes a novel clinical entity and re-frames the definition of the *Virchow node.*

The definition of the Virchow node, as it relates to anatomical and medical terminology, warrants discussion. Indeed, based upon this report and, in addition to the literature in general, left-sided, right-sided, as well as concurrent bilateral lymph node metastatic enlargement is possible. Thus, *Virchow node*, which is classically defined as an enlarged left-sided lymph node resulting from metastasis, may confuse since any supraclavicular lymph nodes may be enlarged by secondary tumors, regardless of laterality. The authors, therefore, suggest that the terminology of so-called *Virchow nodes* must include specificity with regard to laterality (i.e., left-sided Virchow node, right-sided Virchow node, bilateral Virchow nodes) or the eponymous terminology be abandoned in favor of non-eponymous and more anatomically descriptive terms and phrases (e.g., left- and right-sided enlarged supraclavicular lymph nodes due to metastasis).

From an anatomical perspective, bilateral involvement of supraclavicular lymph nodes is an unusual finding for several reasons. First, it is generally regarded that the right lymphatic duct drains the right upper extremity, the right side of the head and neck, and the right upper thorax, whereas the thoracic duct drains everything else. Second, the end node of the thoracic duct (i.e., the node that would become the Virchow node) is uncommon in general. Mizutani et al.^14^ found the node in 14% of individuals. There is little information regarding the prevalence of the end node of the right lymphatic duct. Thus, for bilateral Virchow nodes to manifest, an individual would have to be anatomically predisposed by having bilateral end nodes of each lymphatic duct as well as lymphatic bridging between some part of the drainage fields of the lymphatic ducts.

Several important anatomical structures are identified in the vicinity of the Virchow nodes, and thus might be encroached upon or displaced due to nodal enlargement. Such enlargement and displacement could manifest as mass effects. For example, the phrenic nerve was immediately posterior to the Virchow node. Thus, the phrenic nerve might be compressed between an enlarging Virchow node and the anterior scalene muscle. Such a scenario may contribute to phrenic neuropathy.^9^ Unilateral phrenic neuropathy ranges in severity and can be entirely asymptomatic or cause a range of weakness to the ipsilateral hemidiaphragm. In this case, bilateral enlargement of the Virchow nodes can produce bilateral phrenic neuropathy and thus involve the diaphragm globally— a scenario that could manifest as varied degrees of labored breathing. Encroachment of the transverse cervical artery, which also resides immediately posterior to the Virchow node, could decrease blood flow to the trapezius and sternocleidomastoid muscles and promote spasticity / torticollis, hypothetically.

In addition to the phrenic nerve, the brachial plexus lies posterior to the Virchow node within the scalene triangle. Accordingly, it has been posited that a Virchow node might contribute to thoracic outlet syndrome, both neurogenic and vascular. In this case, bilateral thoracic outlet involvement could be easily misinterpreted as resulting from postural alterations, hypertrophic musculature, muscle imbalances, elevated first ribs, and bilateral cervical ribs.

The vagus nerves were also located against the Virchow nodes. Though rare, vagal nerve compression could be considered with regard to the mass effect of a Virchow node. At the location of the Virchow node, the vagus nerve has typically not given the recurrent laryngeal nerve. Thus, encroachment limiting axoplasmic flow through the fibers that contribute to the recurrent laryngeal nerve could cause laryngeal signs / symptoms (e.g., hoarseness).^15^ Bilateral damage could, hypothetically, cause bilateral vocal cord paresis or paralysis and should, therefore, be considered in the etiology of bilateral vocal cord immobility. To underscore the inclusion of bilateral supraclavicular enlargement in the etiology of bilateral vocal cord paresis / paralysis, Benninger et al.^16^ identified bilateral vocal fold immobility as idiopathic in 12.8% of cases. In a scenario of nerve compression from the Virchow node, other vagus nerve-related autonomic signs and symptoms might be expected to occur concomitantly (unlike isolated recurrent laryngeal nerve involvement).

In this case, the cause of death was respiratory arrest secondary to lung cancer. Emphysematous changes were also identified. In such scenarios, hypertrophy of the anterior scalene musculature may further encourage the abovementioned neuropathies.

## CONCLUSION

This report marks a novel case of metastatic spread of small cell neuroendocrine carcinoma manifesting as concurrent bilateral Virchow nodes.
